# Application of CRISPR/Cas9 System in Establishing Large Animal Models

**DOI:** 10.3389/fcell.2022.919155

**Published:** 2022-05-17

**Authors:** Yingqi Lin, Jun Li, Caijuan Li, Zhuchi Tu, Shihua Li, Xiao-Jiang Li, Sen Yan

**Affiliations:** Guangdong Key Laboratory of Non-human Primate Research, Guangdong-Hongkong-Macau Institute of CNS Regeneration, Jinan University, Guangzhou, China

**Keywords:** Large animals, CRISPR/Cas9, Off-target, Mosaicism, Base editing

## Abstract

The foundation for investigating the mechanisms of human diseases is the establishment of animal models, which are also widely used in agricultural industry, pharmaceutical applications, and clinical research. However, small animals such as rodents, which have been extensively used to create disease models, do not often fully mimic the key pathological changes and/or important symptoms of human disease. As a result, there is an emerging need to establish suitable large animal models that can recapitulate important phenotypes of human diseases for investigating pathogenesis and developing effective therapeutics. However, traditional genetic modification technologies used in establishing small animal models are difficultly applied for generating large animal models of human diseases. This difficulty has been overcome to a great extent by the recent development of gene editing technology, especially the clustered regularly interspaced short palindromic repeats (CRISPR)/CRISPR-associated protein 9 (Cas9). In this review, we focus on the applications of CRISPR/Cas9 system to establishment of large animal models, including nonhuman primates, pigs, sheep, goats and dogs, for investigating disease pathogenesis and treatment. We also discuss the limitations of large animal models and possible solutions according to our current knowledge. Finally, we sum up the applications of the novel genome editing tool Base Editors (BEs) and its great potential for gene editing in large animals.

## Introduction

Animal models play an important role in scientific research, including the study of disease mechanisms, medicinal development and the production of agricultural products (McGonigle and Ruggeri, 2014). To create ideal animal models, researchers often genetically modify animals to achieve desirable traits. Gene-modified small rodent models, especially mice and rats, provide a large amount of experimental data and play an important role in the study of disease mechanisms as well as important biology function (Ribitsch et al., 2020). However, these small animal models also have some shortcomings. First, because of considerable differences between small animals and humans in physiological, anatomical, and genomic structures, small animal models are often unable to mimic the disease characteristics of humans, leading to the inability of researchers to fully understand the pathogenesis of diseases. This has also led to the failure of many drugs screened from small animal models in clinical trials (Prabhakar, 2012; Zhao et al., 2019). In addition, small animal models play less roles in agricultural activities, such as the production of animal by-products.

**FIGURE 1:**
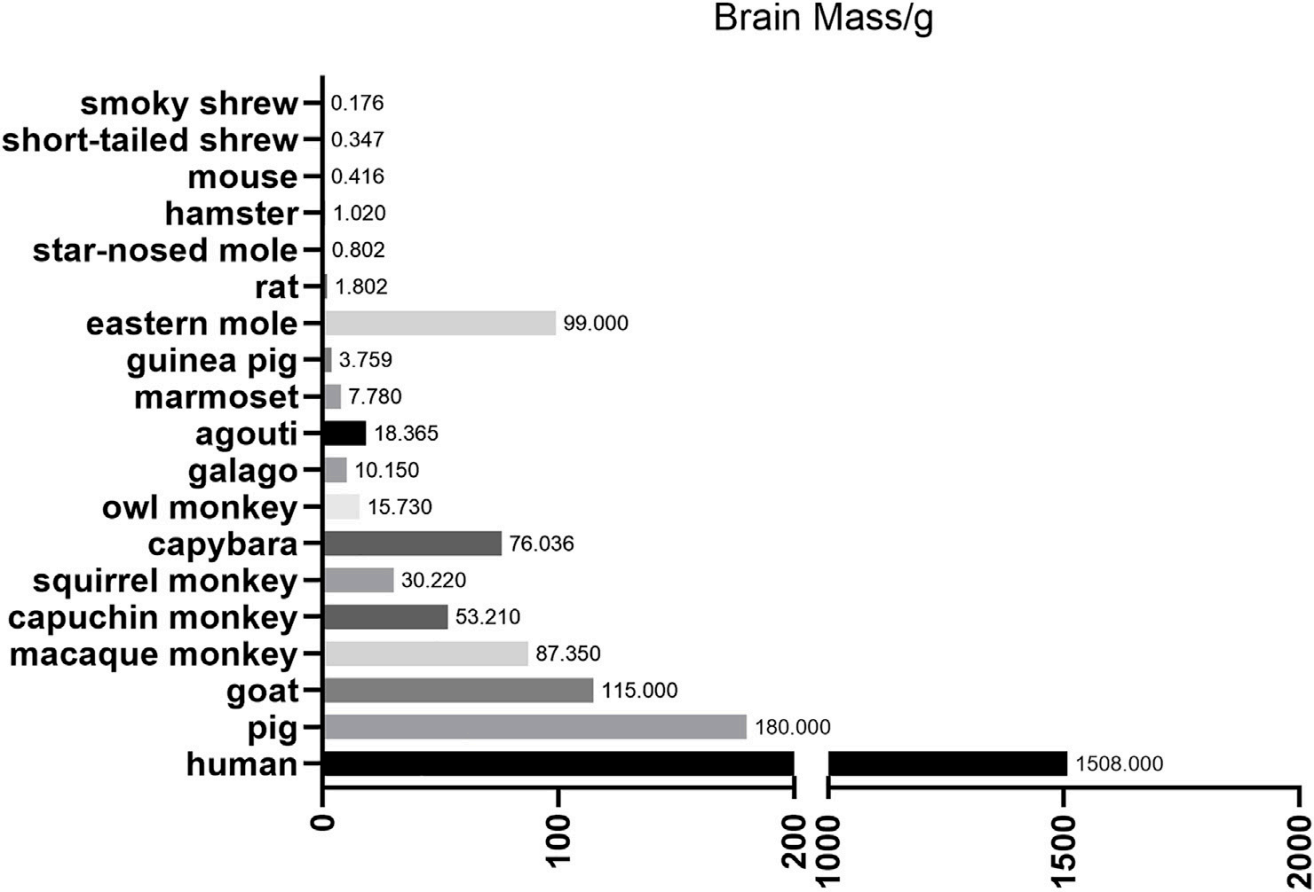
Brain mass for different animals. This figure mainly depicts the brain mass of different large animals and small animals. Notice that large animals, such as pigs and nonhuman primates, are much closer to human beings compared with small animals to some extent.

To overcome these limitations, scientists are increasingly focusing on large animal models (including non-human primates (NHPs), pigs, dogs, goats, and sheep). Large animals represented by NHPs are more ideal animal models for human diseases due to their similarities in genetics, physiology, developmental biology, social behavior and cognition (Shen, 2013). For example, the brain mass of different species, which are depicted in Figure 1, are apparently very different. However, many factors, including the difficulties in genome editing, have limited the establishment of gene modified large animal models.

With the development of gene editing technology in recent twodecades, like zinc finger nucleases (ZFNs), transcription activator-like effector nucleases (TALENs) and CRISPR/Cas9, this difficulty has been overcome greatly. The ZFNs system consists of two components, including DNA-binding zinc-finger protein (ZFP) domain at the amino terminus and the Fok I nuclease cleavage domain at the carboxyl terminus. Each Fok I monomer link with a ZFP to form a ZFN that recognizes a specific site. Under certain conditions, two ZFNs can perform enzyme function of cleavage, leading to double-strand breaks (DSBs), thus mediating DNA site-specific cleavage (Durai et al., 2005; Carroll, 2011). ZFNs have been used in a variety of species, including plants, animal and mammalian cells (Carroll, 2011). However, due to the differences between large animals and small animals, it is still difficult to establish a large animal model using ZFNs(Chen et al., 2016). TALENs are gene-editing tools similar to ZFNs(Christian et al., 2010; Mussolino and Cathomen, 2012; Joung and Sander, 2013). They are also made by two structures: a transcription activator-like (TAL) effector DNA-binding domain and a DNA cleavage domain (a nuclease which cuts DNA strands). Thus, TALENs can also be designed to induce site-specific DSBs to target specific gene sequences (Yoshimi and Mashimo, 2018). Compared with ZFNs, the synthesis and design of TALENs components are simpler, so some large animal models are successfully established by TALENs strategy. For example, in 2014, researchers created methyl CpG binding protein 2 (*MECP2*) Mutant Rhesus and Cynomolgus monkeys using TALENs(Liu et al., 2014).

Although ZFNs and TALENs have greatly improved the efficiency of establishing gene editing animal models, CRISPR/Cas9 is the most popular and effective gene editing method at present. The CRISPR/Cas9 system also consists of a recognition component, small RNAs called single-guide RNAs (sgRNAs), and a cleavage component, Cas9 nuclease. CRISPR/Cas9 can target almost any loosened (non-condensed) part of the genomes through base pairing between sgRNAs and DNA as well as the recognition of protospacer adjacent motif (PAM) sequences (Jinek et al., 2012). Cas9 then cleaves the double-strand DNA at the target site to form DSBs. Subsequently, cells repair DSBs sites by non-homologous end joining (NHEJ) or homology-directed repair (HDR). The repair process can go wrong, resulting in mutations at specific genetic loci. Since the discovery of CRISPR/Cas9, researchers have rapidly implemented a series of optimizations to the system, and applied it in the establishment of gene-editing models of small animal, including mice (Wang et al., 2013), rats (Ma et al., 2014) and zebrafishes (Kim and Kim, 2014). As a result, the CRISPR/Cas9 system has greatly accelerated the research of gene editing large animal models, which is what we mainly discuss in this article.

## The Application of CRISPR/Cas9 in Nonhuman Primate Models

Undoubtedly, the nonhuman primate is the most representative animal that is capable of mimicking the human disease for the similarities in terms of genetics, physiology, developmental biology, social behaviors and cognition. However, it is really difficult to create a nonhuman primate transgenic model compared with small animals, such as the mouse because of various factors including the long breeding cycle and ethical factors. The first transgenic mouse model was built as early as 1974 (Jaenisch and Mintz, 1974), but the first genetically modified monkey model did not appear until 2001 (Chan et al., 2001). Afterwards, in 2008, Yang et al. developed a transgenic model of Huntington’s disease (HD) in a rhesus macaque that expressed polyglutamine (polyQ)-expanded huntingtin protein (HTT) by injecting lentiviral vector into mature rhesus oocytes followed by fertilization through intracytoplasmic sperm injection and embryo transplantation (Yang et al., 2008). This is the first transgenic nonhuman primate disease model, which could already show some features similar to those found in HD patients, including chorea, dystonia as well as nuclear inclusion and neuropil aggregates in the brains. In the later experiment, they used the same transgenic strategy to introduce mutant huntingtin (*HTT*) genes into monkey oocytes, which also expressed exon1 of *HTT* with a 147Q tract (previous models containing a 65–88Q tract and dying soon) in transgenic monkeys (Wang et al., 2008). These HD monkeys display degeneration of axons and neuronal processes, suggesting that the disruption in axons or dendrites can lead to the neuronal degeneration in HD.

The success of transgenic monkey models of human disease and the advances in gene editing technology inspired scientists to establish genome editing monkey models. The generation of a gene-modified monkey via CRISPR/Cas9 was first reported in 2014 (Niu et al., 2014). By coinjection of Cas9 mRNA and sgRNAs into one-cell-stage embryos, researchers successfully achieved precise gene targeting in cynomolgus monkeys. They also showed that this system enabled simultaneous disruption of two target genes (peroxisome proliferator-activated receptor gamma (*PPARG*) and recombination activating gene 1 (*RAG1*) in one step without detectable off-target effects. They also demonstrated that germline transmission could happen in the Cas9-manipulated monkeys by examining gene targeting in gonads and germ cells (Chen et al., 2015a). However, the resulting transgenic monkey exhibited mosaic mutations accompanied by the presence of wild type allele in different tissues, which left an issue of whether the mosaic mutations could influence the function study. Then, Chen et al. used Cas9 to disrupt the dystrophin gene (*DMD*) in rhesus monkeys, which exhibited markedly depleted dystrophin and muscle degeneration seen in early Duchenne muscular dystrophy (DMD) (Chen et al., 2015b), indicating that CRISPR/Cas9 can efficiently generate monkey models of human diseases regardless of inheritance patterns.

Next, some experiments were conducted aimed at eliminating the mosaic mutations. Researchers showed that biallelic gene mutation can be efficiently generated in monkeys by zygote injection with an optimized Cas9/sgRNA combination in one-step (Wan et al., 2015). After optimization and innovation of the approach, Zuo et al. also showed that a single gene or multiple genes can be completely knocked out in monkey embryos by zygotic injection of Cas9 mRNA and multiple adjacent sgRNAs without mosaicism (Zuo et al., 2017). Apart from that, another research indicated that shortening the half-life of Cas9 in fertilized zygotes reduced mosaic mutations and increased its ability to modify genomes in monkey embryos (Tu et al., 2017). Following this approach, they used the Cas9/sgRNA method to disrupt SH3 and ankyrin repeat domains 3 gene (*SHANK3*) in cynomolgus monkeys, which showed altered neurogenesis and disrupted expression of synaptic proteins in the prefrontal cortex, which was not found in the mouse model (Zhao et al., 2017).

Apart from knockout animals, researchers have also attempted to obtain knock-in nonhuman primates in recent years. Yao et al. first established knock-in monkeys by homology-mediated end joining (HMEJ)-based method. However, monkeys generated by this approach showed mosaicism. Therefore, further serial crossbreeding is required to generate complete gene knock-in monkeys (Yao et al., 2018). A similar work to achieve precise *OCT4-*hr*GFP* (octamer-binding transcription factor 4-humanized recombinant green fluorescent protein) knock-in in cynomolgus monkey model was also tried via CRISPR/Cas9-assisted HR (Cui et al., 2018).

However, a genetically modified nonhuman primate is expensive to maintain and requires the facility that is only available to a small number of laboratories. Thus, researchers have tried to establish the modified embryonic stem cells (ESCs) of rhesus monkey using the CRISPR/Cas9 system, which can undergo unlimited self-renewal while maintaining the potential to give rise to all cell types (Zhu et al., 2015; Kobayashi et al., 2019). Using a dual-guide gene targeting approach, another group achieved biallelic deletions in the *CCR5* gene (C-C motif chemokine receptor 5) of cynomolgus macaque embryos (23–37%) (Schmidt et al., 2020).

Although CRISPR/Cas9 system is the most widely used strategy in the creation of large animal models nowadays, there were also nonhuman primate models using different approaches like TALENs(Liu et al., 2014; Liu et al., 2016; Chen et al., 2017). An obvious issue is the safety of the gene-editing in nonhuman primates due to potential off-target mutations. Using whole-genome sequencing to comprehensively assess on- and off-target mutations in previously produced CRISPR/Cas9 editing monkeys (Chen et al., 2015b), researchers found that CRISPR/Cas9-based gene editing is active on the expected genomic sites without producing off-target modifications in other functional regions of the genome, suggesting that the CRISPR/Cas9 technique could be relatively safe and effective in modeling genetic disease in nonhuman primates (Wang S. et al., 2018; Luo et al., 2019).

CRISPR/Cas9 editing monkeys have already shown better phenotypes in human diseases than mouse models despite a limited number of successfully established models (Seita et al., 2020; Yang et al., 2021; Yin et al., 2022). Yang et al. generated PTEN-induced kinase 1 (*PINK1*, whose mutations cause early-onset Parkinson’s disease (PD) mutant monkeys by targeting two exons in the *PINK1* gene (Yang et al., 2019a; Yang et al., 2019b), which showed remarkable neuronal loss in the cortex, substantia nigra and striatum. However, neuronal loss was not reported in *Pink1* KO mice (Kitada et al., 2007; Dawson et al., 2010) or pigs (Zhou et al., 2015; Wang et al., 2016b). Similar results were confirmed in acute monkey models created by CRISPR/Cas9 system (Li et al., 2021; Sun et al., 2022; Yang et al., 2022). In another example, Kang et al. achieved dosage-sensitive sex reversal, adrenal hypoplasia critical region, on chromosome X, gene 1 (*DAX1*) knockout in the monkey, which could recapitulate the phenotypes of human adrenal hypoplasia congenita (AHC) and hypogonadotropic hypogonadism (HH).

Nonhuman primate models of human diseases were also used for developing therapies. Tu et al. found that abnormal behaviors and brain activities of autism spectrum disorder (ASD) in the previously established monkey models (Zhao et al., 2017) were alleviated by the antidepressant fluoxetine treatment (Tu et al., 2019). This finding demonstrated that the genetically modified non-human primate can be used for translational research of therapeutics for ASD, pointing out that the nonhuman primate models of human disease have a great potential for clinical research like drug tests.

## The Application of CRISPR/Cas9 in Pig Models

The pig is also an important animal with several unique features that make it a promising alternative animal model (Prather et al., 2003). Pigs are model animals that are also close to humans (Meurens et al., 2012). Pigs and humans are extremely similar in terms of anatomy, physiology and biochemical metabolism (Roura et al., 2016). Pigs have the advantages of early sexual maturity, short reproductive cycle, high number of offspring per litter (Zou et al., 2019). Moreover, the recent development of somatic cell nuclear transfer (SCNT) technology and genome editing technology has made it possible to generate genetically modified large animals efficiently (Yang et al., 2014). The first genome editing pigs were generated in 1985 using pronuclear DNA microinjection in zygotes (Hammer et al., 1985). With the development of CRISPR/Cas9 technology, the speed of building genome-edited pig models has been greatly accelerated.

Hai et al. first showed that zygotes microinjection of the CRISPR/Cas9 system can efficiently generate genome-modified pigs in one step (Hai et al., 2014), and the mutations can be transmitted into the germline efficiently. Different researchers then reported that single- or double-gene targeted pigs can be effectively achieved by using the CRISPR/Cas9 system combined with SCNT, which avoids mosaic mutation and detectable off-target effects (Whitworth et al., 2014; Zhou et al., 2015). Also, the modification of multiple genes like triple gene-targeted pigs was feasible to be generated (Wang et al., 2016b).

Apart from knockout pigs, CRISPR/Cas system is also used to establish knock-in models that mimic human diseases. Yan et al. used CRISPR/Cas9 to insert a large CAG repeat (150 CAGs) into the endogenous pig *HTT* gene in fibroblast cells and employed the SCNT to generate a HD KI pig model expressing full-length mutant HTT at the endogenous level (Yan et al., 2018), whose brains presented severe and preferential neurodegeneration in the medium spiny neurons like HD patients. Other examples include genes or large fragment knock-in pig models (Ruan et al., 2015; Li G. et al., 2020).

Based on these successful trials in establishing knockout or knock-in animals using CRISPR/Cas9 system, a number of pig models that could recapture the features of human diseases have been created, such as 5-hydroxytryptamine (5-HT) deficiency (Li et al., 2017b), complement protein deficiency (Zhang W. et al., 2017), cardiovascular disease (Huang et al., 2017), cancer (Kang et al., 2016), type II collagenopathy (Zhang et al., 2020), and HD (Yan et al., 2018).

On the other hand, the pig is one of the most important livestock in the agriculture industry. Genetically modified pigs may offer distinct features, such as increased mass of muscle and resistance to the pathogen. As a result, researchers have made great efforts to improve the mass of muscle by using CRISPR/Cas9 system to disrupt genes that hinder the hypertrophy of muscle (Wang K. et al., 2015; Wang et al., 2017; Zou et al., 2018; Liu et al., 2019; Li R. et al., 2020) and improve the resistance to virus (Xie et al., 2020).

Apart from this, the pig is considered to be an important resource of donor organs for transplantation because of the growing demand in the xenotransplantation. A key problem after xenotransplantation is the pig-to-human immunological compatibility. Therefore, a great deal of genome editing pigs has been established to eliminate antigens leading to immunological rejection in human (Petersen et al., 2016; Chuang et al., 2017; Gao et al., 2017; Wu et al., 2017; Joanna et al., 2018). Another problem is the risk of cross-species transmission of porcine endogenous retroviruses (PERVs). Therefore, researchers inactivated all of the PERVs in a porcine primary cell line and generated PERV-inactivated pigs *via* SCNT and CRISPR/Cas9 system (Yang et al., 2015; Niu D. et al., 2017; Yue et al., 2021). These efforts were aimed to make the clinical usage of pig organs safer. Recently, a series of stunning reports have provided the first results showing the feasibility of transplanting organs from transgenic pigs into humans, including the kidney and the heart (2022; Porrett et al., 2022), which marked a great breakthrough in the clinical application.

## The Application of CRISPR/Cas9 in Sheep and Goat Models

Sheep and goats have also become important model animals in biomedical research due to their suitable size and short gestation period. Like pigs, sheep and goats also play an important role in agricultural and pharmaceutical field for their meat, milk, fiber, and other by-products. Han et al. reported the successful one-step generation of gene knockout sheep using a one-step zygote injection of the CRISPR/Cas9 system by targeting the myostatin (*MSTN*) gene (Zhengxing et al., 2014), which demonstrated the feasibility of gene targeting in sheep using the CRISPR/Cas9 system at the first time. At the same year, Ni et al. showed for the first time that the CRISPR/Cas9 mediated genome editing can be efficiently accomplished in goats (Ni et al., 2014) and the single-gene knockout fibroblasts were successfully used for SCNT and resulted in live-born goats harboring biallelic mutations.

Apart from the knockout strategy based on the aberrant DNA repair to generate frameshifting insertion-deletion mutations (indels), whether this genomic engineering technique involving HR can be used to introduce defined point mutations is another question. Subsequently, Niu et al. reported a G→A point mutation in the growth differentiation factor 9 (*GDF9*) gene that has a large effect on the litter size of cashmere goats successfully (Niu et al., 2018). Moreover, Wu et al. succeeded in integrating an exogenous t*GFP* (turbo*GFP*) gene into targeted genes in frame with high efficiency (Wu et al., 2016), which was the first gene knock-in sheep via CRISPR/Cas9 system. Another research specifically inserted the thymosin beta 4 (*Tβ4*) gene into the goat *CCR5* locus (Li X. et al., 2019), which also provided an example for the establishment of knock-in goats models.

Sheep and goats have been used as interesting models in biomedical research. Compared to experimental rodents, sheep and goats offer the advantage of being more suitable in mimicking human diseases due to their similar size and anatomy. There have already been some examples of genome editing sheep or goats by CRISPR/Cas9 system to mimic human disease. Fan et al. created the first sheep model of human disease of cystic fibrosis (CF) generated by CRISPR/Cas9 mediated disruption of the cystic fibrosis transmembrane conductance regulator (*CFTR*) gene (Fan et al., 2018). The newborn *CFTR* ^−/-^ sheep developed severe disease phenotypes consistent with CF pathology in humans, like pancreatic fibrosis, intestinal obstruction, and substantial liver and gallbladder disease reflecting CF liver disease that is evident in humans. Another study reported for the first time the generation of otoferlin (*OTOF*) gene disrupted sheep, which provided a model allowing better understanding and development of new therapies for human deafness related to genetic disorders (Menchaca et al., 2020). Additionally, Williams et al. have also reported an interesting sheep model, recapitulating human hypophosphatasia (HPP, a rare metabolic bone disease) by applying CRISPR/Cas9(Williams et al., 2018). In this study, a single point mutation in the tissue-nonspecific alkaline phosphatase gene (*ALPL*) was introduced. Thus, the generated gene-edited lambs accurately phenocopied human HPP, providing a useful large animal model for the study of rare human bone diseases. The results of these reports corroborate the great potential of the CRISPR/Cas9 system to generate gene-edited sheep or goats that recapitulate human diseases (Kalds et al., 2019).

Similar to pigs, the sheep or the goat is also one of the most important livestock in agriculture industry, which urges scientists to reform its traits via genome editing from different aspects according to physical demand. Sheep and goats could provide us with donor organs for xenotransplantation by serving as the host for the growth of human organs. As a result, Vilarino et al. created *PDX1* ^−/−^ (pancreatic and duodenal homeobox protein 1) fetus lacking a pancreas, which provided the basis for the production of gene-edited sheep as a host for interspecies organ generation (Vilarino et al., 2017). *BMPR-IB* (bone morphogenetic protein receptor type IB, also known as FecB) is a key candidate gene for the genetic control of sheep reproductive performance. Researchers created loss-of-function mutations in the sheep *BMPR-IB* by using the CRISPR/Cas9 system, leading to an increase in ovulation rate and consequently larger litter size (Zhang et al., 2017c). Ma et al. established an *AANAT/ASMT* (aralkylamine N-acetyltransferase/acetylserotonin O-methyltransferase) transgenic animal model constructed with CRISPR/Cas9 system, which served as the mammary gland bioreactor to produce melatonin-enriched milk in the sheep (Ma et al., 2017) or in goats (Zhou et al., 2017; Tian et al., 2018). Another research showed that CRISPR/Cas9-mediated loss of fibroblast growth factor 5 (*FGF5*) activity could promote the wool growth and, consequently, increase the wool length and yield in sheep (Li et al., 2017a; Hu et al., 2017) or in goats (Wang et al., 2016a), and similar research was performed in order to change the coat color of sheep and goats (Zhang et al., 2017b).

To meet the growing demand for the meat product of sheep and goats, many experiments are aimed at improving the yield and quality of the meat production of sheep or goats, most of which were conducted by hindering the muscle producing genes in sheep (Crispo et al., 2015; Niu Y. et al., 2017; Zhang Y. et al., 2018) or in goats (Wang X. et al., 2015; Guo et al., 2016; Zhang J. et al., 2018; Wang X. et al., 2018; He et al., 2018), like *MSTN*. This strategy was also used in the pig.

## The Application of CRISPR/Cas9 in Dog Models

The dog is also a typical species used in scientific research, though there are not many labs focusing on the topic of CRISPR/Cas9 system editing dogs. Zou et al. demonstrated for the first time that a single injection of Cas9 mRNA and sgRNA corresponding to a specific gene into zygotes, combined with an auto-embryo transfer strategy, can efficiently generate site-specific genome-modified dogs (Zou et al., 2015). Their team also generated the apolipoprotein E (*APOE*) deficient dogs via similar strategy 3 years later (Feng et al., 2018).

The most exciting breakthrough about gene edited dogs related to CRISPR/Cas9 system happened in the year of 2018. Researchers of Olson lab working with dogs successfully fixed a genetic glitch that causes DMD by further damaging the DNA. They used adeno-associated viruses (AAV) to deliver CRISPR/Cas9 gene editing components to four dogs, which allowed the mutated gene to again make a key muscle protein and greatly alleviated the disease (Amoasii et al., 2018). The feat-achieved for the first time in a large animal-raised hope that such genetic surgery could 1 day prevent or treat this crippling and deadly disease in people, which created a great interest in the field (Cohen, 2018; Duan, 2018; Wasala et al., 2019; Mata Lopez et al., 2020).

## Discussion

### Limitations of CRISPR/Cas9 System in Large Animal Models

Above all, the progress of genome editing large animal models is accelerated tremendously by CRISPR/Cas9 system because of many aspects of advantages. First, this system is nearly able to target any locus of the genome in animals theoretically as long as there are PAM sequences near the location (Mali et al., 2013), which really expanded the range of editing genes compared with previous strategy. Next, because the targeting strategy relies on 23 base pair matches, CRISPR/Cas9 can target to virtually any genes in a sequence-dependent manner. As a result, CRISPR/Cas9 can target two alleles to cause a null mutation in the founder animals, which would avoid the procedure of mating heterozygous mutant animals to generate homozygous mutants. This advantage is really critical to large animals because the outcrossing process may take much longer time than mouse models, even several years in nonhuman primates. Third, the CRISPR/Cas9 system can simultaneously manipulate several genes by the co-injection of several sgRNAs with Cas9 into fertilized eggs at the one-cell stage, which makes the establishment of animal models of multigenic disease possible, especially the complex neurodegenerative diseases, such as PD and Alzheimer’s disease (AD). As a result, these models may mimic the genetic mutations and the symptoms in patients in a better way.

Although the CRISPR/Cas9 system has brought great hope for the use of large animal models for studying human diseases, several challenges remain. The first one is the off-targeting effect. When the approximate 23 base pairs that enable the specific cutting of Cas9 match other areas of the genomic DNA, nonspecific editing may happen and cause off-targeting mutations. Although the off-target events can be diluted over generations in small animals with short breeding times, this strategy is infeasible for large animals like monkeys because their sexual maturation usually requires 4–5 years (Niu et al., 2014). To eliminate or reduce the side effects of off-target mutations, truncated guide RNAs in the CRISPR/Cas9 system can be used to improve the specificity of Cas9 nucleases or paired nickases (Fu et al., 2013; Sakuma et al., 2014). On the other hand, the use of bioinformatic screening to search for unique genomic targets and the use of paired Cas9 nickases can also reduce off-targets (Tu et al., 2015).

The second problem is the mosaicism in CRISPR/Cas9-mediated genome editing, which means the presence of more than one genotype in one individual. The mechanisms of the mosaicism are still unclear, and there may be several causes. It is possible that the translation of Cas9 mRNA to produce an active enzymatic form is delayed until after the first cell division, and this delay may play a major role in genetic mosaicism. The mosaicism problem may also result from the prolonged expression of Cas9 mRNA. Alternatively, differential DNA repair and non-homozygous recombination activities in zygotes and divided embryonic cells can also influence genetic mutation rates and mosaicism (Tu et al., 2015). In a word, the CRISPR/Cas9 system can continuously target and cleave genes at different stages of embryonic development in different ways, as a result, leading to mosaicism of the introduced mutations. Although the mosaicism is an undesired result in genome editing animals, there may be several advantages, which include enabling animals to survive beyond the lethal phase when manipulated genes are essential to animals. In addition, mosaic animals help us better understand dosage effects of genes on developmental defects, especially those which may mimic human congenital disorders (Zhong et al., 2015). However, the mosaicism caused by CRISPR/Cas9 is undesirable in most cases. The biggest problem of chimeric large animals is that the mutation may be difficult to be transmitted into the offspring (Oliver et al., 2015) because of the long breeding cycle of large animals especially nonhuman primates. There are several possible strategies to reduce the mosaicism. The first way is to speed up the editing process by the introduction of CRISPR/Cas9 components in an appropriate format [Cas9/sgRNA ribonucleoprotein (RNP)] and concentration into very early pronuclear stage zygotes by using electroporation (Mehravar et al., 2019), so the CRISPR/Cas9 system will work at the earliest stage of zygotes. Secondly, as described above, shortening the longevity of Cas9 in combination with embryo splitting to eliminate the delayed function of Cas9 also makes a difference (Tu et al., 2017). The third strategy is to use germline modification. In this way, genetically-modified somatic cells can be used as nuclear donors for SCNT into enucleated germ cells. In another way, generally targeted gene edited spermatogonial stem cells (SSCs) can be used as donors for transplantation into testis directly.

The last problem is the efficiency of the CRISPR/Cas9 system. According to the previous study, the efficiency of gene targeting with CRISPR in large animals (like nonhuman primates) is more variable and lower than that in mice (Chen et al., 2016). Therefore, precise gene editing technologies need to be further improved, to increase the efficiency of gene targeting and the rate of homozygous mutation by using new Cas protein [like Cpf1 (Cas12a)] and new systems (like base editing system).

### Base Editing in Large Animals and Prime Editing

**FIGURE 2:**
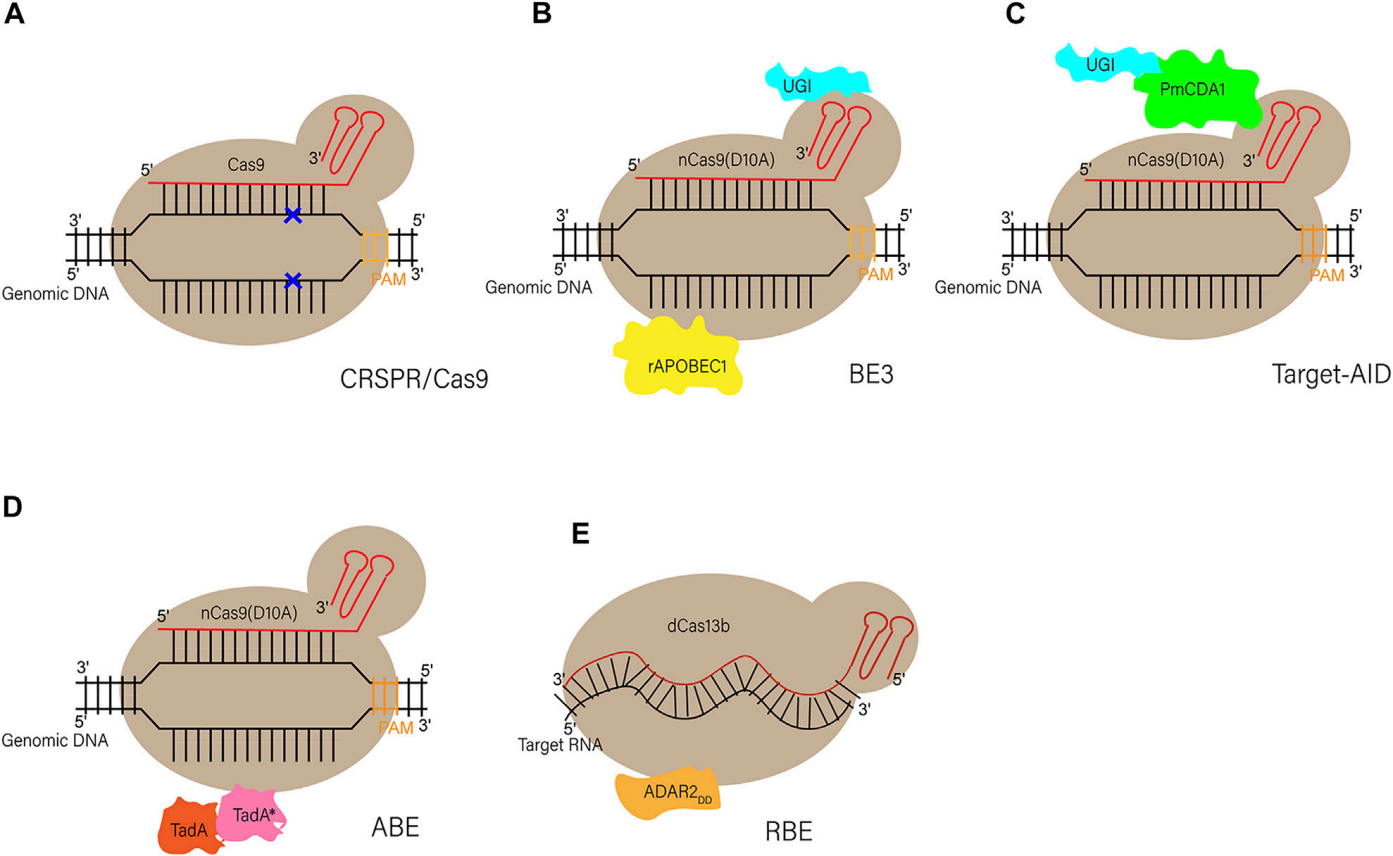
Schematic diagram of CRISPR/Cas9 and different BEs **(A) Schematic diagram of CRISPR/Cas9.** Through base pairing between sgRNAs and DNA as well as the PAM sequence, Cas9 can recognize and cleave the double-strand DNA at the target site to form DSBs **(B,C) Schematic diagram of two original base editors (CBEs).** BE3: a SpCas9 nickase (D10A) is linked to a rat cytidine deaminase (rAPOBEC1) through the N terminus, and to a uracil glycosylase inhibitor (UGI) at the C terminus. Target-AID: the C terminus of SpCas9 nickase (D10A) is linked to both cytidine deaminase from *Petromyzon marinus* (PmCDA1) and UGI **(D) Schematic diagram of adenine base editor (ABEs).** ABEs: fusion of artificially evolved DNA adenine deaminase (TadA^*^-TadA) with SpCas9 nickase (D10A) generates ABEs (TadA, wildtype *Escherichia* coil tRNA adenosine deaminase; TadA^*^, mutated TadA) **(E) Schematic diagram of RNA base editor (RBEs).** RBEs: catalytically dead Prevotella sp. Cas13 (dCas13b) is tethered with deaminase domain of human“adenosine deaminase acting on RNA” (ADAR2_DD_) to form RBEs.

Although the CRISPR/Cas9 system has been used to establish gene editing models in multiple species, it is more likely to induce random indels through error-prone NHEJ rather than the error-free HDR during gene editing (Kim and Kim, 2014), which makes indels more likely to occur at the editing site than single-nucleotide substitutions. In addition, DNA sequencing results show that point mutations, not indels, cause the vast majority of human genetic diseases (Yuan et al., 2020), which suggests the importance of the newly developed gene-editing tool base editing in establishing animal models of human disease. Base editing is a gene editing tool developed in recent years, which can lead to gene mutations through changes in a single base pair (Komor et al., 2016; Nishida et al., 2016; Gaudelli et al., 2017). At the genome level, BEs can achieve all four kinds of single base transition, including C to T, G to A, A to G, and T to C (adenine (A), cytosine (C), guanine (G), and thymine (T)). There are several basic base editors, include cytosine base editors (CBEs), adenine base editors (ABEs) and RNA base editors (RBEs). CBEs is capable of converting the base pair C-G to into T-A, while ABEs can achieve the transition from A-T into G-C. RBEs are able to achieve the conversion of A to Inosine (I) in the level of RNA (Molla and Yang, 2019) (the structure of these BEs are depicted in Figure 2).

Although there are only several years after the invention of base editing, scientists have used it to achieve gene editing successfully in many small animals and plants, such as mouse (Ryu et al., 2018), rat (Ma et al., 2018), rabbit (Lee et al., 2018; Liu et al., 2018), zebrafish (Zhang Y. et al., 2017), rice (Hua et al., 2018) and wheat (Li C. et al., 2018).

Apart from these, there are also some examples of gene editing large animal models created by BEs. In the 2018, a group demonstrated the BE3 (one kind of CBEs)-mediated base editing can induce nonsense mutations in the goat *FGF5* gene. They further characterized the phenotypic and genetic changes to investigate the consequence of base pair editing, and provided strong supporting evidence that the BE3 induced off-target mutations were rare at genome-wide level (Li G. et al., 2019). These successful attempts in goats opened up unlimited possibilities of genome engineering by base editing in large animals. Inspired by this, researchers also created BE3-mediated sheep by co-injection of a BE3 mRNA and guide RNA aiming at the *SOCS2* (suppressor of cytokine signaling 2) gene in the next year (Zhou et al., 2019).

By using BE3 to target the *TWIST2* (twist-related protein 2) gene [responsible for the ablepharon macrostomia syndrome (AMS) in human)] and the tyrosinase (*TYR*) gene [the causal gene for oculocutaneous albinism type 1 (OCA1)], researchers created a gene editing pig model successfully in the same year of the first base editing goats (Li Z. et al., 2018), which mimicked the phenotypic characteristics of human diseases well. The group of Liangxue Lai also achieved efficient base editing for several genes in pigs by combining CBEs with SCNT in the next year, including *DMD*, *RAG1* (recombination activating gene 1), *RAG2* (recombination activating gene 1) and *IL2RG* (interleukin 2 receptor subunit gamma) (Xie et al., 2019). Another group also achieved precise base conversion in three genes {[*GGTA1* (glycoprotein alpha-galactosyltransferase 1), *B4GALNT2* (beta-1,4-N-acetyl-galactosaminyltransferase 2), and *CMAH* (cytidine monophospho-N-acetylneuraminic acid hydroxylasegenes)]} in pig genome (Yuan et al., 2020). As for NHPs, researchers generated the first Hutchinson-Gilford progeria syndrome (HGPS) monkey model by delivering a BE mRNA and guide RNA (gRNA) targeting the *LMNA* (lamin A/C) gene via microinjection into monkey zygotes, and the typical HGPS phenotypes including growth retardation, bone alterations, and vascular abnormalities confirmed the reliability of this model (Wang et al., 2020). Since the appearance of BEs, this tool has been applied in some species of large animals, like NHPs, goats, sheep and pigs. It is really convenient to establish disease models or improve the traits in large animals by base editing for its ability of precise single base pair editing.

Although base editing has played a great role in precise genome editing, it is still unable to achieve all base conversions. To solve this problem, researchers created the prime editing. This system consists of a Cas9 nickase (H840A mutation) fused to a reverse transcriptase domain and a modified sgRNA, named prime editing guide RNA (pegRNA). The basic principle is to use nCas9 to nick the non-target strand at the target location, and then use reverse transcriptase to generate the required sequence using the template RNA. Then the FEN1 endonuclease is able to excise the sequence called flap during this process and contributes to the genome repairing (Anzalone et al., 2019; Caso and Davies, 2022). Although this technique has only been reported in the establishment of small animal models (Liu et al., 2020), it is of great significance for the study of diseases caused by various base mutations in large animal models because it can mediate almost all types of base conversions.

### Prospects and Challenges

Large animal models can be used in many areas including disease pathogenesis investigation and pre-clinical research. Due to the lack of ESCs from large animals, it has been difficult to use traditional gene targeting technology to establish large animal models of human diseases. Nowadays, the development of precise genome editing tools especially the CRISPR/Cas9 system has greatly advanced this field. Many genome-edited large animals have been created including knockout or knock-in that cover almost all types of currently used experimental animals, such as nonhuman primates, pigs, sheep, goats and dogs. Even so, there are still many limitations in the establishment of large animal models, which may involve the inadequate gene targeting efficiency, mosaicism, and off-targeting. Many strategies and optimized components of the system have been brought up to reduce these drawbacks or to improve efficiency.

**FIGURE 3:**
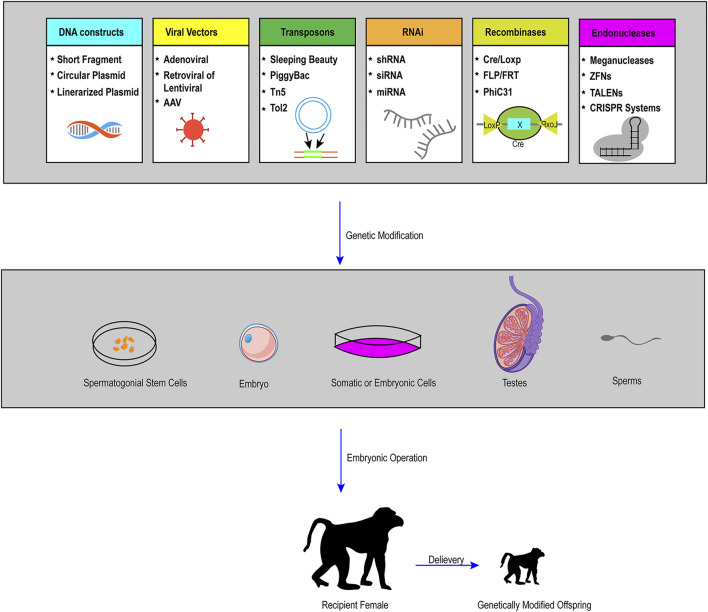
Schematic representation of practical and possible pathways of genetic modification in large animals. To achieve the generation of live founders with desired genetic modifications (pronuclear injection and nuclear transfer are the two primary procedures), the first step is to conduct gene manipulations in a variety of cells or organs, including somatic cells, embryonic cells, embryos, spermatozoa, SSCs and other targeted organs by the use of many tools, such as viral vectors, recombinases, transposons, RNA interference (RNAi), and endonucleases. Through a series of embryonic operations, such as nuclear transfer, genetically modified cells or embryos can produce genetically modified offspring.

The BEs recently provide us with a new tool in the establishment of large animal models with the rapid development and optimization of this new system. Although there are only few successful examples in large animals, the BEs greatly expanded the scope of this area promisingly because the vast majority of human genetic diseases are induced by point mutations. On the other hand, all kinds of other technologies, like somatic cell nuclear transfer, genome editing of SSCs, and tetraploid complementation, have played more and more important roles in the establishment of rodents or even large animal models nowadays. Therefore, the combination of the BEs or optimized CRISPR/Cas9 components with other platforms previously described will make precise genome modification in large animals more efficient and easier (the procedure of gene modified large animal models is depicted in Figure 3).

In clinical research, it is a promising direction to use gene therapy to treat inherited human diseases. The FDA has approved some gene therapy products for patients, such as patients with B-cell precursor acute lymphoblastic leukemia (ALL). Before applying the strategy on patients, evaluating the efficacy and safety using genetically modified animal models that can mimic the characteristics of human disease is necessary, which further underscores the importance of the establishment of genome editing large animal models. Xenotransplantation is another promising direction, as the shortage of human organs is a common problem, and model animals can provide a sufficient supply of organ products. Genetic modification of donor animals can eliminate many problems, especially the immunological compatibility, and increase the probability of success during xenotransplantation. The recent success of transplanting organs from transgenic pigs into the humans is a stunning breakthrough in this field, which will in turn lead to the development of gene-editing large animal models.

In summary, with the development and application of precise genome editing tools represented by CRISPR/Ca9 system, a great number of large animal models will be established (the examples of genome-edited large animals described in this article is listed in Table 1). The improvement will create ideal animal models that are more similar to the human, benefit the study of the mechanisms of human diseases, and make important contribution to the clinical application.

**TABLE 1:** Examples of genome-edited large animals described in this article.

Species	Genes	Editing type	References
NHPs	*RTT*, *MECP2*	KO	Liu et al. (2014)
	*PPARG*, *RAG1*	M-KO	Niu et al. (2014)
	*DMD*	KO	Chen et al. (2015b)
	*TP53*	KO	Wan et al. (2015)
	*ARNTL*, *PRRT2*	M-KO	Zuo et al. (2017)
	*PINK1*, *ASPM*	KO	Tu et al. (2017)
	*SHANK3*	KO	Zhao et al. (2017)
	mCherry	KI	Yao et al. (2018)
	hr*GFP*	KI	Cui et al. (2018)
	*MECP2*	KO	Chen et al. (2017)
	*PINK1*	M-KO	Yang et al. (2019b)
	*LMNA*	BE	Wang et al. (2020)
Pigs	*VWF*	KO	Zhang et al. (2017a)
	*CD1D*, *CD163*, *EGFP*	M-KO	Huang et al. (2017)
	*PRKN*, *DJ-1*, *PINK1*	M-KO	Wang et al. (2016b)
	*HTT*	KI	Yan et al. (2018)
	Large transgene cassette	KI	Li et al. (2020a)
	*F9*	KI	Chen et al. (2021)
	Large transgene cassette	KI	Ruan et al. (2015)
	*TPH2*	KO	Li et al. (2017b)
	*C3*	KO	Zhang et al. (2017a)
	*APOE*, *LDLR*	M-KO	Huang et al. (2017)
	*RUNX3*	KO	Kang et al. (2016)
	*MSTN*	KO	Wang et al., (2015a)
			Wang et al., 2017
			Li et al. (2020b)
	*IGF2*	KO	Liu et al. (2019)
	*FBX O 40*	KO	Zou et al. (2018)
	p*RSAD2*	KI	Xie et al. (2020)
	p*ULBP1*	KO	Joanna et al. (2018)
	*PDX1*	KO	Wu et al. (2017)
	*GGTA1*, *CMAH*	M-KO	Gao et al. (2017)
	*GGTA1*	KO	(Petersen et al., 2016
			Chuang et al. (2017)
	*GGTA1*, *BGALNT2*, *CMAH*	BE	Yuan et al. (2020)
	*TWIST2*, *TYR*	BE	Li et al. (2018b)

species	Genes	Editing type	References
goats	*MSTN*	KO	Ni et al., (2014)
			Guo et al., 2016
			He et al., 2018
			Wang et al. (2018b)
	*CDF9*	PM	Niu et al. (2018)
	*Tβ4*	KI	Li et al. (2019b)
	*SCD1*	KO	Tian et al. (2018)
	*BLG*	KO	Zhou et al. (2017)
	*FGF5*	KO	Wang et al. (2016a)
	*MSTN*, *FGF5*	M-KO	Wang et al. (2015b)
	*FAT1*, *MSTN*	KO/KI	Zhang et al. (2018a)
	*FGF5*	BE	Li et al. (2019a)
sheep	t*GFP*	KI	Wu et al. (2016)
	*CFTR*	KO	Fan et al. (2018)
	*OTOF*	KO	Menchaca et al. (2020)
	*ALPL*	PM	Williams et al. (2018)
	*PDX1*	KO	Vilarino et al. (2017)
	*BMPR-IB*	KO	Zhang et al. (2017c)
	*AANAT*, *ASMT*	KI	Ma et al. (2017)
	*FGF5*	KO	Hu et al., (2017)
			Li et al. (2017a)
	*ASIP*	KO	Zhang et al. (2017b)
	*MSTN*	KO	Crispo et al., (2015)
			Rao et al., 2016
			Zhang et al. (2018b)
	*BC O 2*	KO	Niu et al. (2017b)
	*SOCS2*	BE	Zhou et al. (2019)
dogs	*MSTN*	KO	Zou et al. (2015)
	*APOE*	KO	Feng et al. (2018)

The table lists genes that have been changed by the way of KO, M-KO, PM or BE or KI. Abbreviations: KO, knockout; M-KO, multiplex knockout; KI, knock-in; PM, point mutation (by HDR); BE, base editing.
